# Contrast diversity patterns and processes of microbial community assembly in a river-lake continuum across a catchment scale in northwestern China

**DOI:** 10.1186/s40793-020-00356-9

**Published:** 2020-04-25

**Authors:** Xiangming Tang, Guijuan Xie, Keqiang Shao, Yang Hu, Jian Cai, Chengrong Bai, Yi Gong, Guang Gao

**Affiliations:** 1 Taihu Laboratory for Lake Ecosystem Research, State Key Laboratory of Lake Science and Environment, Nanjing Institute of Geography and Limnology, Chinese Academy of Sciences, Nanjing, 210008 China; 2 University of Chinese Academy of Sciences, Beijing, 100049 China

**Keywords:** Microbial community assembly, Species and functional diversity, Deterministic and stochastic processes, Salinity, Biotic interactions, Lake Bosten

## Abstract

**Background:**

Microorganisms in rivers and lakes are essential for nutrient recycling in aquatic ecosystems. Understanding the ecological processes shaping microbial communities is of crucial importance for aquatic microbial ecology and biogeography. However, the diversity of microorganisms and the forces that control this diversity are poorly understood. This is particularly true within the framework of the river-lake continuum in arid regions.

**Results:**

Using a whole catchment-sampling effort, we explored biogeographical patterns and mechanisms of microbial community (bacteria and archaea) assembly within the catchment of the largest inland once freshwater lake (Lake Bosten) in China. Water samples from headstream tributaries, the mainstream of the River Kaidu to downstream Lake Bosten were characterized using amplicon sequencing of 16S rRNA genes. Higher α-diversity was found in mainstream of River Kaidu and in the tributaries compared with Lake Bosten. And the microbial community composition was also significantly different between the lake and its connected river habitats. Canonical correspondence analysis demonstrated that salinity and total suspended solids were the most important environmental factors shaping the community variations. Overall, pure environmental and pure spatial factors explained 13.7 and 5.6% of the community variation, respectively, while 32.0% of the variation was explained by combined environmental and spatial variables. These observations suggested that spatially structured environmental variations mainly shaped the microbial biogeography in this region. Both deterministic and stochastic processes influenced the microbial community assembly in river and lake habitats, and the stochastic pattern was particularly pronounced for microbiome in river habitat. Co-occurrence network analysis revealed more abundant and complicated correlations among frequently occurred taxa in lake habitat compared with the river habitat, implying that ecological multispecies interactions (e.g., competition) shaped lake microbial community structures.

**Conclusions:**

Our findings demonstrate an ecological succession along the river-lake continuum of microbial communities across the largest inland once freshwater lake basin in China, and highlight the effects of spatially structured environmental factors on regional microbial *β*-diversity and species interactions on local community assembly.

## Background

Inland aquatic ecosystems are facing increasing pressures from various anthropogenic impacts and/or climate changes in their watersheds [1]. One of the major problems is freshwater salinization [2]. For example, Lake Bosten located in the arid central Asia, which used to be the largest inland freshwater lake in China, has evolved to be an oligosaline lake during the past 60 years with the salinity increased from 0.38 g/L to 1.5 g/L [3]. As the most abundant, diverse and functionally important organisms on Earth [4], microorganisms in aquatic ecosystems can response to environmental changes quickly, and have a key role in ecological processes including the biodegradation of pollutants that impact water quality [5].

Streams and rivers link terrestrial, lotic and lentic systems with their lake counterparts and supply numerous ecosystem services such as material transport, biogeochemical nutrient cycling and habitats for biota (including microbes). They also provide potable water for public consumption and supply water for irrigation and industry, which is important in arid and semi-arid regions (occupying nearly 40% of the Earth’s land surface). Rivers and lakes have traditionally been studied as separate entities, particularly in studies of aquatic microbial ecology. Spatial and temporal patterns of bacterial diversity and biogeography have been demonstrated in several large rivers, such as the Thames River [6], the Danube River [7] and the Yangtze River [8], as well as a few lakes or freshwater reservoirs [9–11]. However, streams and rivers change constantly as they move from headwaters to the downstream lakes, thus rivers and lakes can only truly be understood as a continuum [12–14]. At present, there is a knowledge gap concerning the relationships between microbial communities in lakes and their input rivers in arid and semi-arid regions. How and to what extend do freshwater streams and rivers affect microbial communities in their salinized downstream lakes have still not been comprehensively investigated. This limits our understanding of ecosystem structures and functions and hindering effective predicting the responses of lake ecosystems to environmental change.

Disentangling the drivers of microbial community structure and function in response to environmental change are a central issue in microbial ecology [15]. With the increasing pressures by anthropogenic activities and climate change, understanding ecological mechanisms that govern the specific local adaptations of microbiomes at both community assembly and function levels are of a surge of interest [16]. Currently, it has been accepted that both niche-based deterministic and neutral-based stochastic processes occur simultaneously during the assembly of local communities [15, 17–21]. Niche-based deterministic theories suggest that environmental filtering, biotic interactions and interspecific trade-offs largely determine patterns of species diversity and composition. In contrast, stochastic theories emphasize the importance of chance colonization, demographic randomness and ecological drift [22]. Based on this perspective, various theoretical models and practical algorithms quantifying the importance of both deterministic and stochastic processes have been developed and applied [23–29].

The species-abundance distribution (SAD), which is defined as the distribution of abundances across species in a community, is one of the most important patterns in macroecology and biogeography [4, 30]. Since the 1930s, more than 20 models that predict the SAD have been developed [31]. Among these models, niche-based broken-stick (BS) [32] and geometric-series (GS, i.e., niche preemption) [33], neutrality-based Volkov [34], as well as purely statistical Poisson lognormal (PLN) and log-series models have been frequently used in predict macrobial SADs. While the SAD is central to macroecology and biodiversity theory, microbiologists have largely neglected the connection of the SAD to the mechanisms of microbial community assembly [4].

While the temporal and spatial variations of microbial community composition in Lake Bosten and its upstream tributaries have been examined recently [35, 36], little is known about the differences in microbial diversity and the mechanisms of underlying community assembly of river-lake continuum between Lake Bosten and its linked streams and rivers. In this study, we used the Lake Bosten watershed (Additional file 1: Fig. S1) as a model ecosystem to investigate the microbial diversity and the community assembly mechanisms in both river and lake systems simultaneously based on null model [28, 29], SAD models and co-occurrence network analysis [37, 38]. We hypothesized that (i) the river system has higher species diversity than the lake system because of spatial heterogeneity and closer link with terrestrial ecosystems; (ii) deterministic assembly is vital to form microbial communities in lake system because of the filtering effects of salinity and biotic interaction. This study is the first attempt to explore the microbial diversity patterns and the community assembly mechanisms in a river-lake continuum in the arid central Asia. Therefore, it makes fundamental contribution to the mechanism understanding for a predictive microbial ecology in river-lake continuum under the circumstances of a future increasing tendency of lake salinization in arid and semi-arid regions.

## Results

### Microbial diversity, community structure and taxonomy

**Fig. 1:**
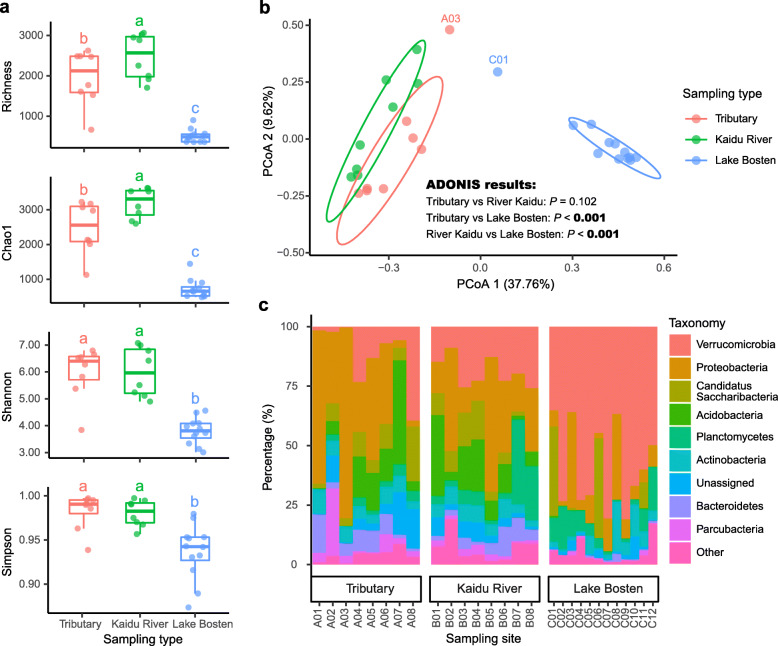
Diversity and taxonomy of microorganisms in the upstream tributaries, River Kaidu and Lake Bosten. **a** Comparison of α-diversity indexes of microbial communities. Diversity indexes were calculated using subset of 23,965 reads per sample. Horizontal bars in the box plots indicate median proportional values. Lower and upper edges of the boxes represent the approximate 1st and 3rd quartiles, respectively. The upper and lower whiskers extend to data no more than 1.5 times the interquartile range from the upper edge and lower edge of the box, respectively. Kruskal-Wallis test was performed to examine differences among the three sampling types. Different lower-case letters indicate significant differences (*P* < 0.05) among sampling types. **b** Unconstrained PCoA with Bray–Curtis distance showing that the microbial community in Lake Bosten separate from those in River Kaidu and the upstream tributaries using *adonis* analysis. Ellipses cover 80% of the data for each sampling type. **c** Phylum-level distribution of microbial communities of each sampling site

After demultiplexing, quality filtering, denoising, removing of chimera, chloroplast and low abundance of unique sequences (< 10 reads), we generated a total of 1,231,890 high-quality reads (average length = 263 bp), averaging 43,996 reads per sample, which were classified into 5049 amplicon sequence variants (ASVs) across the 28 samples, taken from Lake Bosten catchment. For the comparison of *α*- and *β*-diversity among different sampling types (i.e., upstream tributaries, the River Kaidu and Lake Bosten), same number of reads in each sample were chosen at random based on the smallest sample (23,965 reads). The rarefaction curves of richness approached an asymptote after 80% reads were calculated, which indicated that our sequencing depth was sufficient (Additional file 2: Fig. S2). Species richness and Chao1 in the mainstream of the River Kaidu (mean = 2476 and 3195, respectively) were significantly higher than those of in upstream tributaries (mean = 1955 and 2477, respectively; *P* < 0.05) and in Lake Bosten (mean = 510 and 726, respectively; *P* < 0.001) (Fig. 1a). There were no significant differences (*P* > 0.05) of Shannon diversity and Simpson index in upstream and mainstream of the River Kaidu. However, both of them were significantly higher than those in Lake Bosten (*P* < 0.01). Spearman correlation analysis showed that the four microbial *α*-diversity indices were significantly positively correlated to total suspended solids (TSS), total phosphorus (TP) and dissolved oxygen (DO), and significantly negatively correlated to total dissolved solids (TDS), water temperature (WT), pH and dissolved organic carbon (DOC) (Additional file 3: Fig. S3).

Unconstrained principal coordinates analysis (PCoA) of Bray–Curtis distance was performed to evaluate *β*-diversity among different sampling types (Fig. 1b). The results showed that the first two axis explained 47.4% of the microbial community variation. Adonis revealed that microbial communities in Lake Bosten was significantly separated from those in upstream and mainstream of the River Kaidu (*P* < 0.001), while no significant differences were recorded between the latter two communities (*P* > 0.05, Fig. 1b).

For the taxonomy, among the 5049 ASVs only 64 ASVs with 4298 reads were assigned to archaea, representing 0.35% of the total reads (Additional file 4: Table S1). All of these 64 ASVs were presented in the upstream tributaries and mainstream of the River Kaidu, while only 17 (26.6%) of them were presented in Lake Bosten with relative low abundance (accounted for 0.9% of the total archaea reads). *Euryarchaeota* and *Thaumarchaeota* were the predominant phylum among the kingdom of archaea, accounting for 40.6 and 57.8% of the total archaea’s ASVs, respectively. In the phylum of *Thaumarchaeota*, 97.3% of the ASVs belong to genus of *Nitrososphaera*.

Overall, the reads belong to the kingdom of bacteria were classified and grouped under 21 phylum-level taxonomic groups. A dramatic shift in bacterial community composition was observed from upstream tributaries to Lake Bosten (Fig. 1c; Additional file 5: Fig. S4). The most common phyla of bacteria were *Proteobacteria* (average 34.8%), *Acidobacteria* (12.4%), *Verrucomicrobia* (11.5%), Candidatus *Saccharibacteria* (8.4%), *Bacteroidetes* (6.7%), *Actinobacteria* (6.2%) and *Parcubacteria* (6.0%) in upstream tributaries; while the most dominated bacterial phylum in the mainstream of the River Kaidu was also *Proteobacteria* (25.6%), followed by *Verrucomicrobia* (19.1%), *Acidobacteria* (13.1%), *Planctomycetes* (10.2%), Candidatus *Saccharibacteria* (7.4%), *Actinobacteria* (5.7%) and *Bacteroidetes* (5.3%). In Lake Bosten, however, the predominant bacterial phyla were *Verrucomicrobia* (58.9%), *Proteobacteria* (12.1%), Candidatus *Saccharibacteria* (7.6%), *Planctomycetes* (7.6%) and *Actinobacteria* (5.4%). At class-level, the most common bacteria were *Spartobacteria*, *Betaproteobacteria*, *Verrucomicrobiae*, *Alphaproteobacteria*, *Planctomycetia*, *Actinobacteria*, *Acidobacteria* Gp6 and *Bacilli* across all sampling types; while at genus-level, the most common bacteria were *Spartobacteria* genera incertae sedis, *Saccharibacteria* genera incertae sedis, Gp6, *Limnohabitans*, *Parcubacteria* genera incertae sedis, *Polaromonas*, *Rhodopirellula* and *Sphingorhabdus*.

### SAD pattern, microbial community and predicted function between river and lake habitats

**Fig. 2:**
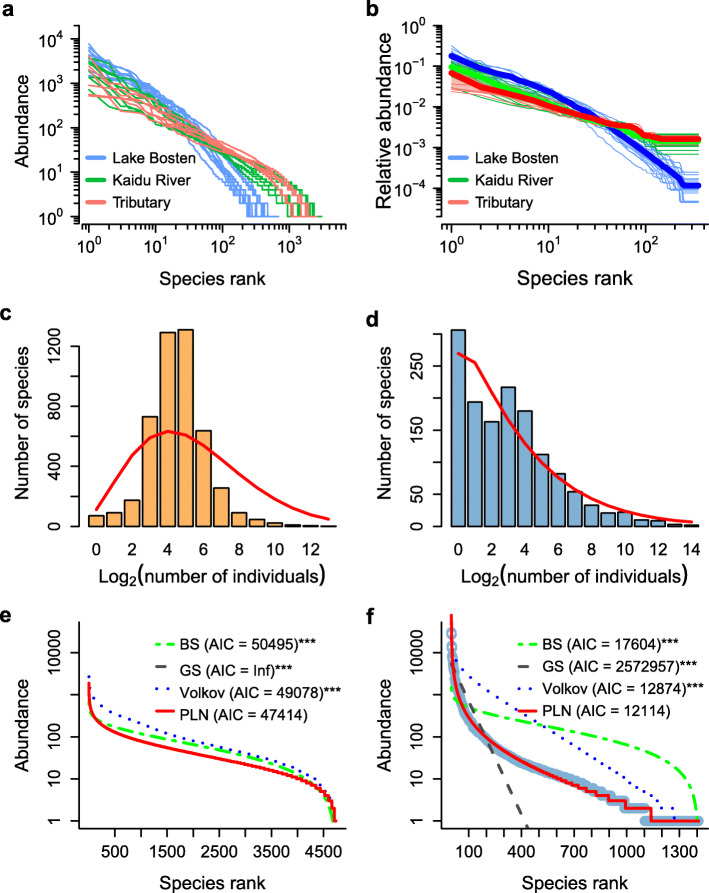
The patterns of species-abundance distributions (SADs) in microbial communities of this study. **a** A rank abundance distribution plot for all samples. **b** Normalized rank abundance distributions (NRADs) with the lowest species (350) sample for samples from upstream tributary (red), River (green) and Lake Bosten (blue). Bold lines and their shaded regions are mean NRADs and the 95% confidence intervals, respectively. SADs of grouped data (binned) from river (**c**, combination of upstream tributary and River Kaidu) and lake (**d**, Lake Bosten) habitats with the predicted values linked as red lines. Observed and fitted SADs for the river **e** and lake **f** microbial communities. Observed values are shown as open circles and fitted models are shown as lines. BS, GS, Volkov and PLN represent broken-stick, geometric-series, Volkov’s neutral community distribution and Poisson log-normal distribution models, respectively. The model was rejected when Kolmogorov-Smirnov (*K-S*) test *P* < 0.05 and the smaller the Akaike’s information criterion (*AIC*) value, the more robust the fit. ^***^K-S test *P* < 0.001. In both habitats, the PLN clearly provide a superior fit

Species rank-abundance curves for all samples were plotted using raw richness data and normalized data (Fig. 2a, b). The data set exhibited a very similar pattern between tributary and the River Kaidu samples, which were quite different from Lake Bosten samples. Because of similar community compositions (Fig. 1b) and SAD pattern (Fig. 2a, b) between samples from upstream tributaries and mainstream of the River Kaidu, we combined tributary and river samples representing river habitat. Samples A03 and C01 (in the river mouth of Lake Bosten) were excluded for subsequent analysis because of abnormal communities (Fig. 1b). In total, 26 samples were divided into two groups representing river (*n* = 15) and lake (*n* = 11) habitats, respectively. SAD plot of lake habitat exhibited a very strong dominance by low abundance species (rare species: reads < 10) compared with river habitat with the relative proportions of rare ASVs of 54 and 12%, respectively (Fig. 2c, d). Lake habitat has a much higher proportion of rare species, but also higher individuals of common species. We estimated SADs of river and lake habitats using BS, GS, Volkov and PLN models (Fig. 2e, f). Both niche-based BS and GS models as well as neutrality-based Volkov model were rejected by Kolmogorov-Smirnov (*K-S*) test, which suggested that none of deterministic and stochastic processes could explain the microbial assembly solely in river and lake ecosystems. However, the statistical PLN model fitted the SADs very well for both river and lake habitats.

**Fig. 3:**
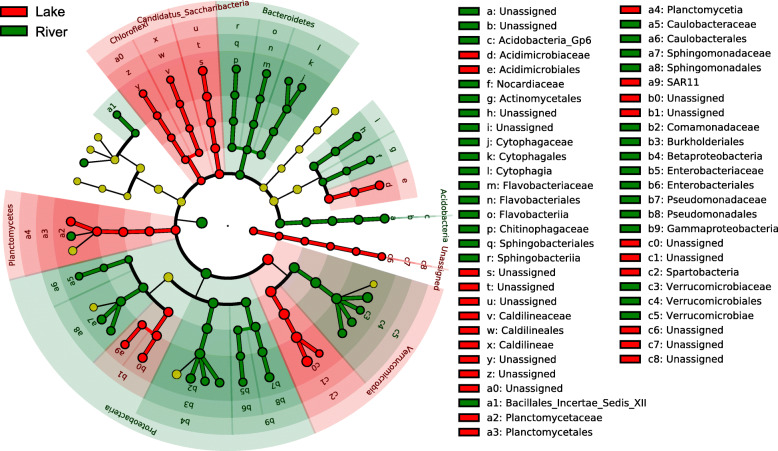
LEfSe results showing the taxonomically differences of microbial communities between rive and lake habitats. Green circles represent those microbes significantly enriched in river habitat and red circles represent those microbes significantly enriched in lake habitat, respectively, whereas the yellow circles represent the taxa with nonsignificant differences between the two types of habitats. Statistics was performed using log linear discriminant analysis (LDA) with LDA > 3.5 and *P* < 0.05 after correction by the Benjamini and Hochberg false discovery rate (FDR) test. The diameters of the circles are proportional to relative abundance

To designate the specialized microbial lineages for each type of habitat, linear discriminant analysis effect size (LEfSe) was implemented with default parameters in order to find microbial groups with statistical differences at different taxonomic levels. A total of 97 microbial taxa were found to be significantly different between river and lake habitats (Fig. 3). Generally, the mean proportions of *Bacteroidetes* (from phylum to genus levels) and *Proteobacteria* (including *β*- and *γ*-*Proteobacteria*) were significantly higher in river habitat, while *Chloroflexi* (from phylum to genus levels), Candidatus *Saccharibacteria* (from phylum to genus levels), *Planctomycetes* (from phylum to family levels), *Verrucomicrobia* and a kind of unassigned microbes were significantly higher in lake habitat. Notably, in the phylum of *Verrucomicrobia*, *Verrucomicrobiae* (from class to genus levels) were enriched in river habitat, while *Spartobacteria* (from class to genus levels) were enriched in lake habitat. In addition, the ubiquitous SAR11 clade (a9) of *Alphaproteobacteria* was also found to be enriched in lake habitat (Fig. 3).

Overall, microbial communities between river and lake habitats shared considerable proportions of ASVs (i.e., 1727 shared ASVs), representing 36.1 and 86.5% of the entire ASVs in river and lake habitats, respectively. While the top 10 ASVs in river habitat were presented in lake habitat with low relative abundances, the top 10 ASVs in lake habitat were seldom appeared in river habitat (Additional file 6: Table S2). For example, we found the top 10 ASVs in lake habitat accounted for 49.95% of the entire microbial community, while they represented only 0.06% of the total reads in river habitat. In Lake Bosten, 9 of the top 10 ASVs belong to the class of *Spartobacteria* and the other one belongs to the clade of SAR11.

**Fig. 4:**
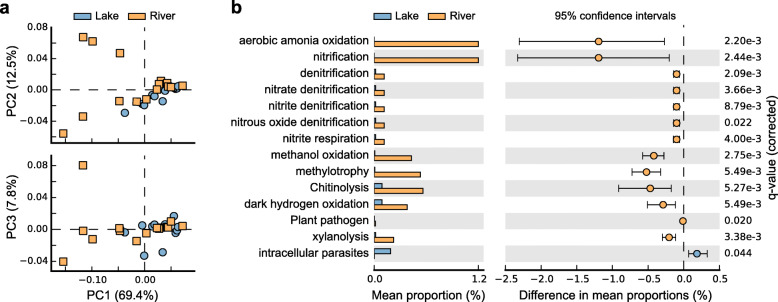
Putative function profiles of microbial communities in river and lake habitats. **a** PCA plots comparing the whole function profiles. **b** Functional categories differing significantly between the river and lake habitats

Functional annotation of ASVs revealed a rich repertoire of metabolic function groups in the river and lake microbial communities. In total, 989 out of 5049 ASVs (19.6%) were assigned to at least one functional group, representing 44 out of 90 functional groups in the functional annotation of prokaryotic taxa (FAPROTAX) 1.1 database. There were 44 annotated functional groups in river habitat while only 32 of them were founded in lake habitat (Additional file 7: Fig. S5), indicating higher functional diversity in river habitat compared with lake habitat. Principal component analysis (PCA) of functional profiles showed no distinct separation between river and lake habitats, but the functional variation in river habitat was much higher than that in lake habitat (Fig. 4a). Among the putative functions, chemoheterotrophy and aerobic chemoheterotrophy were the most abundant groups in both habitats. Functions related to methanogenesis (contributed by the phylum of *Euryarchaeota* in Archaea), aerobic ammonia oxidation (contributed by the genus of *Nitrososphaera* in *Thaumarchaeota*) and cellulolysis (contributed by the genus of *Blastocatella* in *Acidobacteria*) were only founded in river habitat (Additional file 7: Fig. S5). Using White’s non-parametric *t*-test, we found that the functional groups of nitrogen related functions, methanol oxidation, methylotrophy, Chitinolysis, dark hydrogen oxidation, plant pathogen and xylanolysis were significantly enriched in the river habitat, while the mean proportion of intracellular parasites was higher in lake habitat (Fig. 4b).

### Effects of geographic distance on microbial community composition in river and lake habitats

**Fig. 5:**
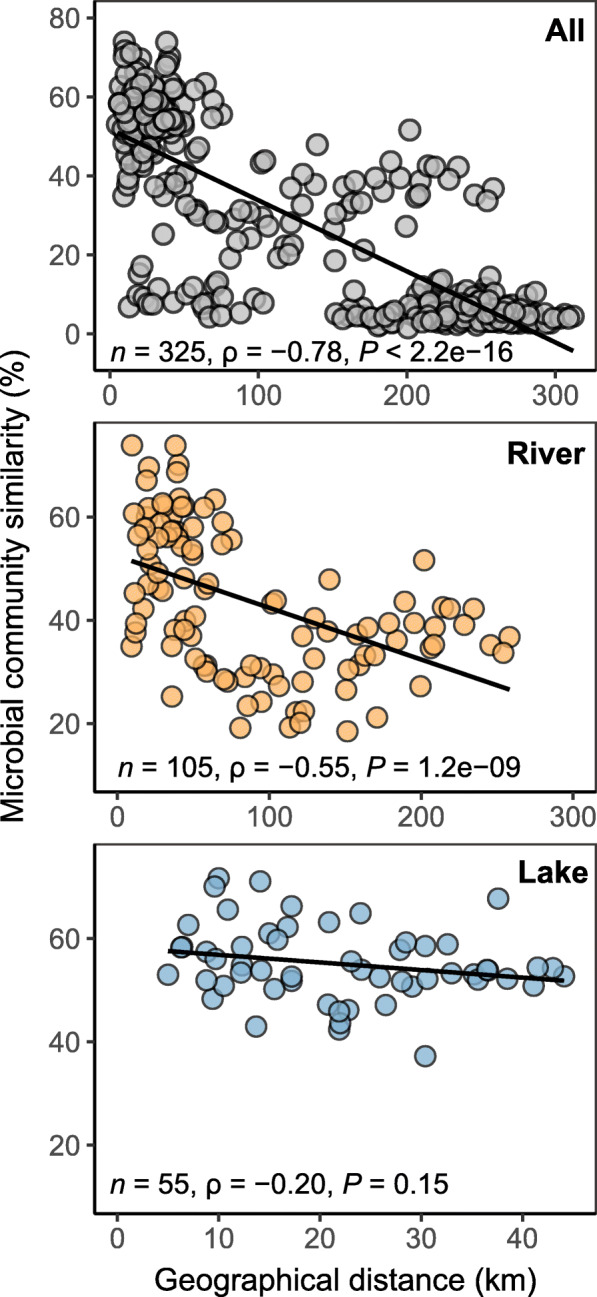
Spearman’s rank correlations between the Bray–Curtis similarity of microbial communities and geographical distance in all samples, river habitat samples and lake habitat samples. Note: *n*, ρ, and *P* refer to the number of comparisons, rank correlations and statistical significances, respectively

Our results revealed that the similarity in microbial community composition decreased with increasing geographic distance (Fig. 5). The Spearman’s correlation between the Bray–Curtis community similarity and geographic distance showed significantly negative correlations for the microbial community with a correlation coefficient (ρ) of − 0.78 and − 0.55 (*P* < 0.001) for the entire and river habitat communities, respectively. Within lake habitat, however, microbial community similarity did not exhibit significant relationship with the geographic distance (ρ = − 0.20, *P* = 0.15; Fig. 5). The distance decay patterns suggest that geographical distance could be of importance in structuring the microbial community assembly and determining the spatial dissimilarity between different sites along the River Kaidu to Lake Bosten, but not within Lake Bosten.

### Influential factors on microbial community compositions

Mantel test demonstrated that the microbial community composition showed significant correlations with environmental parameters (Mantel *r* = 0.887, *P* < 0.001) and geographical distance (Mantel *r* = 0.768, *P* < 0.001). In addition, environmental matrix also showed significant correlation with geographic matrix (Mantel *r* = 0.749, *P* < 0.001), indicating a strong interaction between spatial scale and environmental variables.

**Fig. 6:**
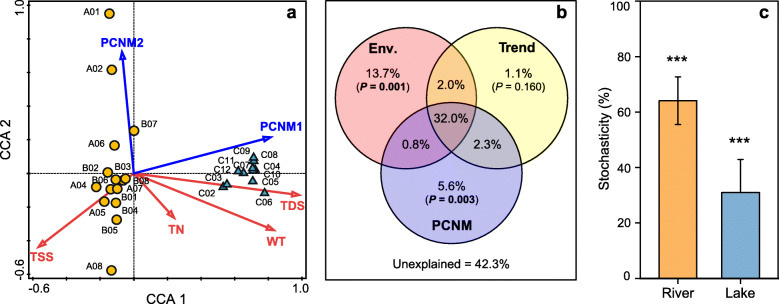
Drivers of microbial community composition. **a** CCA ordination showing the bacterial community composition in relation to significant local environmental variables and regional geographical factors (*P* < 0.05). TDS, total dissolved solids; WT, water temperature; TN, total nitrogen; TSS, total suspended solids. Both the two CCA axes are statistically significant (*P* < 0.05). **b** Venn diagram presenting the variation partitioning results for microbial communities by environmental variables (Env.) and the spatial factors including linear trend and PCNM variables. The fraction values displayed are computed from adjusted R-squares. **c** Relative importance of stochastic mechanism on community assembly in river and lake habitats. The significance differences of the actual communities from those of the related null expectation are indicated as ***P < 0.001 based on PERMANOVA test

The forward selection procedure in canonical correspondence analysis (CCA) revealed that the variation of microbial communities among Lake Bosten catchment are related to four environmental variables, i.e.*,* TDS, TSS, WT and total nitrogen (TN) (Fig. 6a; Additional file 8: Table S3). TDS was found to be the most important factor in structuring the microbial community assemblages, explaining 28.4% of the total variations solely. Principal coordinates of neighbor matrices (PCNM) analysis showed that 57.7% of the variation in the microbial communities could be explained by environmental parameters, linear trend and spatial scale (Fig. 6b). Pure environmental and pure spatial factors accounted for 13.7 and 5.6% of the microbial community variation, respectively, while 32.0% could be attributed to the interaction between environmental and spatial variables, indicating the strong interactions between them.

### Stochastic process on microbial community assembly

To disentangle the relative importance of stochastic mechanisms from deterministic mechanisms in shaping the microbial community structure, the stochastic ratio (SR) was calculated using the null model analysis. Our result demonstrated that the stochastic processes contributed to considerable portions of the community variations (Fig. 6c). The much higher value of SR on community variation in river habitat (64.1 ± 8.6%) than that in lake habitat (31.0 ± 11.9%) suggested that stochastic process could play more important roles in shaping riverine microbiome.

### Co-occurrence networks of microbial sub-communities in river and lake habitats

**Fig. 7:**
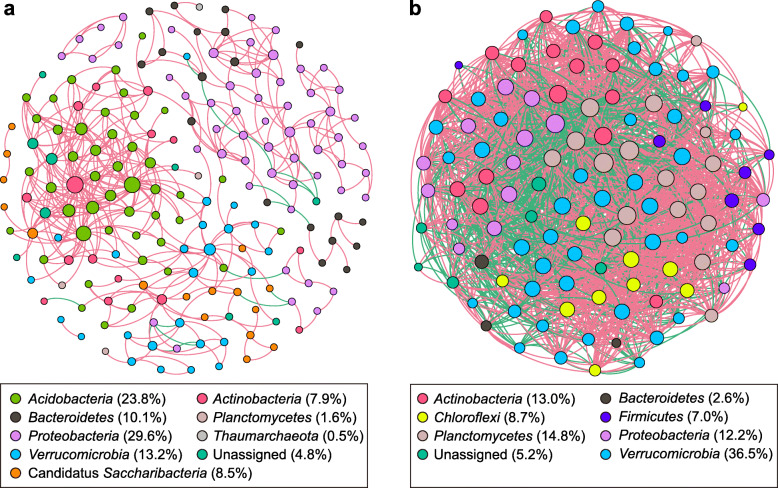
The correlation-based networks of abundant and frequent ASVs (relative abundance > 0.05% and occurred in > 50% samples) for the rive **a** and lake **b** habitats. The size of each node is proportional to the number of connections (i.e., degree), and the nodes are colored according to different phyla. Numbers inside parentheses following names of each phylum represent relative proportion of nodes belonging to the phylum. Red and green edges indicate positive and negative correlations

Co-occurrence networks of microbial communities in river and lake habitats were constructed separately to reveal the ecological interactions among different microbial species. Based on correlation analysis, 189 nodes with 460 edges and 115 nodes with 2104 edges were captured in the networks of river and lake microbial communities, respectively (Fig. 7; Table 1). The higher value of average clustering coefficient (avgCC) and modularity in river and lake networks than those in their related random networks indicate “small-world” properties and modular structure of our constructed networks. It was verified by the value of small-world coefficients (σ) of the river (3.2) and lake (1.4) networks with σ > 1 indicate “small-world” properties [39].

**Table 1:** Topological properties of the abundant (relative abundance > 0.05%) and frequent ASVs (occurred in > 50% samples) co-occurrence networks and their identically sized Erdős-Rényi random networks in different habitats (river vs. lake)

	Empirical network										Random network		
	N	E		Modularity	avgCC	APL	ND	AD	GD	σ	Modularity	avgCC	APL
		Positive	Negative										
River	189	443 (96.3%)	17 (3.7%)	0.653	0.376	7.07	17	4.87	0.026	3.2	0.412	0.056	3.33
Lake	115	1417 (67.3%)	687 (32.7%)	0.211	0.549	1.70	3	36.59	0.321	1.4	0.086	0.397	1.69

N: No. of nodes; E: No. of edges; avgCC: Average clustering coefficient; APL: Average path length; ND: network diameter; AD: Average degree; GD: Graph density; σ: small-word coefficient

σ = (avgCC/avgCC*r*)/(APL/APL*r*) and σ > 1 indicates “small-world” properties, i.e., high interconnectivity and high efficiency [39]. Subscript *r* indicates the properties of the random network

The average degree (AD) and graph density (GD) in the lake network were about 7.5 and 12.3 times, respectively, as high as those in river network (Table 1), indicating more interactions among the species in the lake network. Moreover, the co-occurrence network in river habitat was mostly positively structured (96.3%), indicating ecological mutualistic relationships or cooperation among the riverine microbiome, while much higher proportion of negative correlations among edges was found in the lake network (32.7%), highlighting the effect of competition in lake community assembly.

Most of the nodes in the two networks were associated to seven dominant phyla, i.e., *Proteobacteria*, *Verrucomicrobia*, *Acidobacteria*, *Actinobacteria*, *Bacteroidetes*, Candidatus *Saccharibacteria* and *Planctomycetes* (Fig. 7). Among them, *Proteobacteria* (29.6%) was the most dominant phylum in the riverine network, whereas *Verrucomicrobia* (36.5%) was the most dominant phylum in the lake network. Furthermore, ASVs from the same phyla were more likely to co-occur. Based on topological roles analysis of each ASVs, we found that river network had much stronger clustered topology (modularity). And modularity analysis revealed that all of the ASVs in the lake network were peripherals, while 4 nodes (ASVs) were found to be module hubs and 5 ASVs could be classified as connectors in the river network (Additional file 9: Fig. S6). Among the four module hubs, two of them belonged to *Verrucomicrobia*, the third hub belonged to the genus *Limnohabitans* and the last one was assigned to *Actinobacteria*. Among the five connectors, 4 of them were classified as genus Gp6 of *Acidobacteria*, and the other was *Luteolibacter yonseiensis*, within *Verrucomicrobia* phylum.

## Discussion

### Different microbial community diversities between river and lake habitats

In this study, we found the microbial *α*-diversity in the mainstream of the River Kaidu was much higher than that in Lake Bosten (Fig. 1). There are several possible explanations for this result. On the one hand, the high richness found in the river habitat are likely resulted from the massive immigration of terrestrial, sediment and periphyton microbiome [40–42]. It is well recognized that terrestrial soil normally contains highly diverse microbes that serves as regional microbial pool for its connected aquatic ecosystems [42]. This notion is supported by the fact that all diversity indices were significantly and positively correlated to TSS (Additional file 3: Fig. S3), an indicator of surrounding soil erosion. Shao et al. [43] found that the soil microbial community in this alpine grassland were dominated by *Thaumarchaeota*, *Chloroflexi*, *Acidobacteria*, *Planctomycetes* and *Proteobacteria*, most of which were also enriched in the river habitat in this study. In addition, we found the closest relatives in GenBank of the most abundant ASVs (i.e., ASV_13 and ASV_5) were mostly isolated from soil, periphyton, biofilm or sediment (Additional file 10: Table S4). This indicates a more dynamic coupling between local river community and the surrounding regional metacommunities than the lake habitat does.

On the other hand, the high environmental and spatial heterogeneity among different headwater tributaries and different locations of the main river could attribute different niches for diverse microbes inhabiting. This environmental heterogeneity includes gradients of water temperature (ranged from 4.5 to 17.5 °C), TP (0.064–1.180 mg/L) as well as TN (0.17–2.72 mg/L), which was hardly to be found in the lake habitat (Additional file 11: Fig. S7). Previous researched have proposed that network modules (or clusters) could be interpreted as overlapping niches in which groups of taxa are more densely interconnected than with others [38, 44, 45]. In this study, network topology analysis revealed that the modularity of the river microbial network was notably higher than lake network (Table 1), suggesting more ecological niches in river habitat for diverse microorganisms to perch.

Except for species diversity, we also observed higher functional diversity in river habitat compared with the lake habitat (Fig. 5; Additional file 7: Fig. S5). For example, significant enrichment of genes associated with nitrogen metabolism and methanogenesis were found in the river habitat due to higher abundance of *Nitrososphaera* and *Euryarchaeota* in this habitat. This was consistent with previous results suggesting that small streams and rivers play crucial roles in denitrification and nitrogen uptake, as well as export over nitrogen to downstream lakes [1, 46, 47]. However, it should be noted that the predicted functional profile was limited by the proportion of robust ASVs which can be annotated by FAPROTAX. In our study, there are about 80% ASVs could not be assigned to any functional group, suggesting that massive of unclassified taxa with unknown functions inhabit at this inland aquatic ecosystem [3, 35].

### Different microbial community assembly mechanisms between river and lake habitats

One novel finding of this study is that stochastic process is the main factor controlling the community assembly in river ecosystem, while deterministic process (such as environmental filtering and species interaction) dominated in lake ecosystem (Figs. 6 and 7). In riverine habitat, microorganisms showed strong biogeographic patterns, i.e., distance-decay (Fig. 5), which is evidence for dispersal limitation. Since waterflow has direction, microorganisms in upstream tributaries are likely to be transferred to the mainstream by passive dispersal, which is evidenced by higher *α*-diversity in the mainstream of the River Kaidu compared with upstream tributaries (Fig. 1a). However, microorganisms in mainstream of the River Kaidu are hardly to be transferred back to upstream tributaries, resulting in dispersal limitation. The distance decay of similarity can be caused by either limit to dispersal or by a decrease in environmental similarity with distance. High proportion (32%) of the microbial community variation explained by the interaction between environmental and spatial variables (Fig. 6b) highlight the environmental gradients in shaping the distance decay pattern. Although dispersal can be either deterministic, stochastic, or both [15, 21], potential strong dispersal limitation could be largely viewed as stochastic in river habitat in this study.

Except dispersal limitation, ecological drift may be another important process that contribute the high proportion of stochastic ratio since low density of microorganism was typically found in upstream tributaries (mean = 7.7 ± 8.8 × 10^5^ cells/ml) as well as in mainstream of the River Kaidu (9.4 ± 6.9 × 10^5^ cells/ml) compared with Lake Bosten (13.8 ± 3.5 × 10^5^ cells/ml) (Additional file 11: Fig. S7). It is believed that communities are more susceptible to ecological drift when selection is weak, and the local species abundance is low [22].

In the lake ecosystem, the relative importance of stochastic process on the microbial community variation was small (Fig. 6c), indicating that deterministic process was more important. Local environmental sorting, e.g., salinity, could be one of the main deterministic processes [20]. Salinity has been identified as the most important environmental determinant in shaping microbial communities on global scale [48] and regional or local lakes [9, 49]. In this study, we found that salinity (i.e., TDS) was the most important environmental factor accounted for 28.4% of the total community variations solely (Fig. 6a; Additional file 8: Table S3). Salinity may provide a physiological barrier for some freshwater microbes and favor certain bacteria to thrive [50]. The PCoA plot (Fig. 1b) showed clear separation of microbial community similarity in Lake Bosten from riverine habitat as well as low variations within lake habitat (except the estuary), which consistent with the fact that the open lake constitutes a far more constant and buffered environment than river water, and indicate the existence of an autochthonous community uniquely adapted to the environmental conditions prevailing in this brackish environment. For instance, among the top 10 ASVs in the brackish Lake Bosten, 9 of them belong to class *Spartobacteria* of phylum *Verrucomicrobia* (Additional file 6: Table S2). This is consistent with a report that *Spartobacteria* comprise an important component in the brackish surface water of the Baltic Sea, constituting up to 28.9% of the total bacterial reads [51]. Although *Spartobacteria* could be found in freshwater environment, it seems that members of *Spartobacteria* are more adaptable to brackish habitat, where they are presumably involved in the utilization of phytoplankton-derived organic matter [52]. This fits the observation that significant higher concentrations of chlorophyll-*a* (Chl-*a*) and DOC in the brackish water of Lake Bosten compared with those in river habitat (Additional file 11: Fig. S7). In addition, the fourth most abundant ASV (Additional file 6: Table S2) in lake habitat assigned to the freshwater SAR11 (i.e., LD12) had 100% sequence identity over the 263 bp v4 region of the 16S rRNA gene with the strain LSUCC0530, which was isolated from Lake Borgne with a salinity of 2.39 g/L [53]. It could grow at salinities between 0.36 and 4.7 g/L with the optimal salinity of 1.45 g/L, which is near the mean salinity of Lake Bosten (Additional file 11: Fig. S7). LD12 bacterioplankton are characterized as small cell volumes and are adapted to oligotrophic habitats with obligate aerobic chemoorganoheterotrophic lifestyle. It seemed that Lake Bosten could provide proper environment for bacteria like LD12 to thrive, as reported in previous studies [3, 35]. In summary, the communities in Lake Bosten experiencing salinization process during the last 60 years were much more similar due to niche selection imposed by salinity. The salinization of Lake Bosten is the result of both human activity (such as agricultural reclamation and construction of sluices and pumping stations to change the flow pattern) and regional climate warming [35]. This result highlighted the impact of anthropogenic activities on lake microbial community via the increment of lake salinity in arid area.

Except the high selection pressure by the increased salinity, water residence time (WRT) was expected to be another important local environmental filter shaping the microbial community in Lake Bosten. Niño-García et al. [41] have been shown that bacterial community composition was predominantly structured by hydrology in samples with WRT shorter than 10 days. Moreover, lakes that have WRT > 200 days were characterized by significant dissimilarities in bacterial community composition between the lake water and the inlet [54]. The mean WRT in Lake Bosten is about 970 d [55]. With the long WRT in Lake Bosten, the influence of river communities has impacts only in the river mouth (such as the Site C01, Fig. 1b) through mass effects with large amount of inflow events. In other area of Lake Bosten, however, homogeneous physiochemical environment including much higher salinity applies similar niche for microorganisms differed from river habitat to harbor.

Prior studies that have noted that when WRT is relatively long, many inlet microbial species disappeared or persist in very low abundance, and species sorting appears to be the predominant mechanism shaping the pelagic community within the lake [11, 56]. In line with this, we found much more interactions and competitions among organisms (i.e., species sorting) in the lake community assembly using co-occurrence network analysis (Fig. 7; Table 1). In addition, predation of microbes by protists in the pelagic of Lake Bosten supposed to be more common than that in river habitat since the diversity of microbial eukaryotes in river habitat was much lower than that in lake habitat [57]. With 26 protists taxa and 34 rotifers taxa in Lake Bosten [58], severe competition exclusion and/or predation may partially explain the low species diversity and functional diversity in this lake compared with river habitat (Fig. 1; Additional file 7: Fig. S5). On the other hand, under the condition of limited resources in the surface water of Lake Bosten, biotic interactions (e.g., competition) could maintain multiple species coexistence via differentiating the capacity of microbes to finely partition niche axes (i.e., narrowed niche breadth) with small population sizes (i.e., increased proportion of rare species) [59]. This was supported by the fact that much higher proportion (54%) of rare ASVs was found in lake habitat compared with river habitat (12%) (Fig. 2). The evidence presented here supports the hypothesis that species interactions are extremely important in shaping community assembly in brackish Lake Bosten.

The SAD within a community reflects resource use by the species individually which can be conceptualized in terms of niche or stochastic processes of population dynamics [60]. In this study, the shapes of SADs in river and lake habitats are quite different. Compared with river habitat, in lake habitat a few species were relatively more dominant (higher number of sequences) while many species become proportionally rare. The strong role of salinity (Fig. 6; Additional file 8: Table S3) and geographic factors (Fig. 5) implies that niche processes (e.g., niche division) and stochastic processes (e.g., distance decay) may leave imprints on the SADs in either lake or river habitat. However, the statistical Poisson lognormal, a lognormal model with Poisson-based sampling error, fitted the data better than both the fits from niche-based and neutrality-based models (Fig. 2e, f). This is consistent with Shoemaker et al. [4], who found that the lognormal had provided the most accurate predictions for microbial SADs. This is in contrast to researches from macroorganisms with overwhelmingly log-series distribution [61, 62]. Due to the disparity in sampling scales between microbial and macrobial communities [63], microbial species are generally affected by bewildering multiplicative processes, such as population fluctuations, dimensional axes of the environment, and limited or ‘realized’ niche space by biotic interactions [4, 60]. The fitted Poisson lognormal pattern of SADs may reflect the simultaneous effects of both deterministic and stochastic processes on microbial communities.

## Conclusions

By means of a cross-catchment survey, our results revealed significant differences in both microbial *α*-diversity and functional diversity between lake and its connected riverine ecosystems. Here we showed that dramatically decline of microbial diversity and functions related to nitrogen metabolic and methanogenesis occurred during the transition between river and lake habitats. In addition, we found stochastic processes (such as distance decay and spatial heterogeneity) dominated community assembly in riverine habitat, while deterministic niche-based processes (such as anthropogenic induced lake salinization and biotic interactions) were the leading mechanism controlling assembly in lake habitat. This study is the first attempt to explore the microbial diversity patterns and the community assembly mechanisms in a river-lake continuum in the arid central Asia. We propose that deterministic processes, especially salinity filtering and biotic interactions, may overwhelm the influences of stochastic processes on community assembly in lake ecosystems under the circumstances of a future intensified human activities with increased tendency of lake salinization in arid and semi-arid regions.

## Methods

### Sampling and contextual environmental variables

Lake Bosten (86°40′–87°26′ E and 41°56′–42°14′ N) was previously the largest inland freshwater lake in China. It is located in the lowest area of Yanqi Basin in arid northwestern China [64]. It has a surface area of 950 km^2^ (1046 m above sea level), a maximum depth of 16 m and an average depth of 7 m [35]. The River Kaidu is the sole perennial river supplied about 85% of average annual water inflow runoff (about 3.5 × 10^9^ m^3^) into Lake Bosten. Originated in the snow- and glacier-covered Tianshan mountains, the River Kaidu has a total length of 560 km with numerous branches and a catchment area of 47,900 km^2^. The lake’s watershed lies in the center of the Eurasian continent with an inland desert climate, in which the mean annual precipitation is 64 mm and mean annual evaporation is 1881 mm [65]. Due to anthropogenic activities and climate warming, dramatic changes in water level and salinity during the last 50 years have led to this freshwater lake evolved to oligosaline lake with an average salinity of 1.5 g/L.

Samples were collected from 28 monitoring sites during July 16–21, 2014, including 8 sampling sites from upstream tributaries (A01-A08), 8 sites along the mainstream of the River Kaidu (B01-B08), and 12 sites in Lake Bosten (C01-C12) (Additional file 1: Fig. S1). All sampling sites from upstream tributaries and most sampling sites (B01-B06) from mainstream of the River Kaidu locate in the Bayinbuluke alpine grassland with an elevation ranging from 2385 m to 2950 m and an annual mean temperature of − 4.0 °C [66].

Surface water (top 50 cm) was collected with a 2 L water sampler. Subsamples of 300–500 ml water for 16S rRNA gene analysis were filtered on 0.2 μm pore-size polycarbonate filter (Millipore) using a hand-driven vacuum pump in the field. The filters were stored at − 20 °C in a vehicle-mounted refrigerator during transportation, and subsequently stored at − 80 °C in the laboratory until DNA was extracted. The remaining water samples were preserved at 4 °C and then transported to laboratory for immediate chemical analysis.

### Physicochemical analysis

Water temperature, pH, electrical conductivity (EC), total dissolved solids (TDS), salinity, and dissolved oxygen (DO) were determined in situ using a multi-parameter water quality sonde (YSI 6600 v2, Yellow Springs Instruments Inc., USA). Concentrations of total nitrogen (TN), total phosphorus (TP), total suspended solids (TSS), chlorophyll-*a* (Chl-*a*) and dissolved organic carbon (DOC) were determined in the laboratory according to standard methods [55]. The physicochemical parameters were shown in Additional file 11: Fig. S7.

### Flow cytometry of bacterial abundance

To count total bacterial abundance, a subsample aliquot (10 mL) of each sample was fixed using freshly prepared formaldehyde with a final concentration of 2% for 1 h at room temperature and stored in the dark at 4 °C overnight for the following analysis. An aliquot of 0.5 ml from each sample was stained with SYBR Green I (Sigma-Aldrich, UK) diluted using dimethyl sulphoxide at a final concentration of 1:10000 for 20 min at room temperature in the dark. An addition of 2.5 ml of 1.0 mm diameter beads (Life Technologies, UK) to each sample was used as a calibration and counting standard. Each sample was run for 1 min at a low flow rate (< 1000 events per second) on a FACSJazz flow cytometer (Becton Dickinson) equipped with 488-nm excitation laser according to previous protocol [67]. Bacteria were detected using a combination of side scatter light (related to cell size) vs green fluorescence (FL1, 530/40 nm, due to SYBR Green staining of nucleic acids). Samples were measured in triplicate.

### DNA extraction and Illumina sequencing

DNA was extracted using FastDNA® Spin Kit for Soil (MP Biomedicals) according to the manufacturer’s instruction. The 16S rRNA genes were amplified by polymerase chain reaction (PCR) was performed using the universal primers U789F (5′-TAGATACCCSSGTAGTCC-3′) and U1068R (5′-CTGACGRCRGCCATGC-3′) targeting the V5-V6 region of most bacterial and archaeal 16S rRNA genes [68, 69]. Based on the Silva database (https://www.arb-silva.de/), the coverages of the forward and reverse primer for archaea were 96.6 and 99.2%, respectively, while the coverages of both primers for bacteria was 98.0%.

The PCR amplification was performed using a touchdown program as described previously [36]. Triplicate amplified 16S rRNA genes for each sample were pooled after purification. After quantification of amplicon concentration, equimolar amounts of barcoded amplicons for each sample were sequenced on an Illumina MiSeq PE300 platform by Majorbio Bio-pharm Technology Co. (Shanghai, China).

### Data processing, denoise and taxonomy assignment

Paired-end sequencing reads were merged using FLASH (Fast Length Adjustment of Short reads, v1.2.11) [70]. Adapters and primers were trimmed off all reads using Cutadapt (v1.9.1) [71]. Low quality reads (total expected errors > 1) were discarded using USEARCH (v10.0.240) [72]. Filtered reads were input into VSEARCH (v2.12.0) to generate all the unique sequences and their abundance (abandon unique sequences with abundance < 10). Then, denoise amplicon read including chimera filter were performed using USEARCH’s *unoise3* command [73]. Chimeras were further detected and removed based on SILVA database (release 123). Finally, representative amplicon sequence variants (ASVs) were generated using VSEARCH’s *usearch_global* command with 97% similarity [74]. The representative ASVs were subsequently annotated using the SILVA database to identify the taxonomy of each ASV. The ASVs affiliated with chloroplasts and mitochondria were excluded from downstream analysis. The ASVs are similar with traditional operational taxonomic units (OTUs) except for higher accuracy.

### Microbial diversity and functional annotation

To normalize the sequencing depth of different samples, we randomly selected a subset of 23,965 reads per sample based on the sample with the lowest number of sequences. This resampled ASVs summary table was used for subsequent statistical analyses. Then, α- and β-diversity of the bacterial communities were measured using the USEARCH pipeline (http://www.drive5.com/usearch/manual/pipe_diversity.html). Four indices, i.e., richness, Chao1 (a nonparametric species richness estimator), Shannon index (a combination of richness and evenness) and Simpson diversity were calculated, respectively, to measure the α-diversity [75–77]. Statistical differences of α-diversity indices among upstream tributary, main channel of the River Kaidu and Lake Bosten were performed using Kruskal-Wallis test. A comparison of microbial communities with Bray–Curtis distance in different sampling types was performed by an *adonis*() function in the *vegan* package using R 3.5.3 (https://www.r-project.org) and RStudio 1.1.463 platform.

Functional annotation of taxa was performed using the package FAPROTAX on the normalized ASV table [78, 79]. FAPROTAX is a manually constructed database that maps prokaryotic taxa (e.g., species or genus) to putative functions based on available literature on cultured representatives, which focuses on marine and lake biogeochemistry. In this study, each taxonomically annotated OTU was compared against FAPROTAX_1.1 database (including 7820 annotations and covering 4724 taxa) automatically in a Linux system.

### Comparison of SAD pattern, taxonomy and function between river and lake habitats

To compare the differences of SADs, taxonomy and function between river and lake habitats, samples from upstream tributaries and mainstream of the River Kaidu were grouped together as river habitat while samples from Lak Bosten were grouped together as lake habitat. Then, the BS, GS, Volkov and PLN models were fitted to the SAD of river and lake data, respectively. To test their goodness-of-fit, we used the Kolmogorov-Smirnov (*K-S*) test and Akaike’s information criterion (*AIC*). The model was rejected when *K-S* test *P* < 0.05 and the smaller the *AIC* value, the more robust the fit [62]. The analyses were conducted using R platform with the package *RADanalysis*, *gambin* and *sads*.

Taxonomy profiles from phylum to genus level (summarized from ASVs with > 0.1% relative abundance) between river and lake habitats were compared using linear discriminate analysis (LDA) effect size (LEfSe) [80]. The predicted functional profile from FAPROTAX annotation on the basis of raw ASV tables were compared between river and lake habitats. It is defined as significant when using two-sided White’s non-parametric *t*-test with Benjamini–Hochberg false discovery rate (FDR) *P*-value < 0.05 and LDA > 3.5. The comparisons were visualized on the software STAMP v2.1.3 [81].

### Environmental and spatial factors associated with patterns of microbial community

The distance-decay model was fitted with spatial distance (calculated by geographical coordinates using *SoDA* package in R) and microbial community Bray-Curtis similarity among samples [82]. Mantel tests were carried out using the *vegan* package to examine the Spearman’s rank correlation between the geographic distance matrix and the bacterial community similarity using Bray-Curtis distance matrices with 999 permutations.

The roles of spatial factors were estimated by the method of PCNM [83]. A forward selection procedure [84] was performed to select significant environmental variables and linear trend factors. Then, the variation of the community composition was partitioned between the selected square root transformed environmental variables and the extracted PCNM spatial variables, as well as linear trend factors using a partial redundancy analysis (pRDA) in the *vegan* package [85, 86]. This pRDA allows the total explanations of microbial community variation to be decomposed into fractions that indicate the relative importance of pure environmental variables, pure spatial variables, spatially structured environmental variation (shared fraction) and unexplained variation. Then, a variation partitioning approach (VPA) was used to test the relative importance of environmental variables and spatial factors in structuring microbial communities. Mantel tests were also performed to calculate the Spearman’s rank correlations among environmental Euclidean distance matrix, geographic distance matrix and microbial communities Bray-Curtis distance matrix with 999 permutations using the *vegan* package in R.

CCA was used to explore the significant environmental variables and PCNM spatial variables that associated with microbial community compositions because detrended correspondence analysis (DCA) showed the length of the first axis > 4 [87]. Before the analysis, ASVs data were log (*y* + 1) transformed to reduce the effect of highly abundant ASVs. Environmental variables that produced significant correlation (nonparametric) with microbial community Bray-Curtis similarities were selected as explanatory variables for subsequent CCA analysis. Such variables were square root transformed to improve the distributions before the analysis of forward selection [88]. The significance of the CCA model was tested using ANOVA with 999 permutations.

### Ecological processes govern the microbial community assembly

The null model analysis using abundance-based *β*-diversity matrices [22, 27–29] was further performed to assess the relative importance of deterministic and stochastic processes driving microbial community assembly using the R code described by Zhang et al. [26]. In general, the difference between the observed similarity matrices and the null model expectation was used to quantitatively estimate the strength of stochastic ratio in shaping microbial community variation. And the significance of *P* value was calculated by comparing the observed *F* value with those from 1000 randomized data sets using permutational multivariate analysis of variance (PERMANOVA).

### Network construction and analysis

Microbial interaction is one of the main drivers that contributes to deterministic process of community assembly. In order to gain more insight into the importance of interspecies interaction on community assembly, the species co-occurrence patterns of both river and lake habitats were constructed using network theory [15, 37, 89]. To simplify the dataset (resampled ASVs table), ASVs with relative abundance > 0.05% in river/lake samples were selected. Subsequently, those ASVs only detected in more than 50% of the samples were used for network construction. The network analyses were performed based on the online Molecular Ecological Network Analysis (MENA) pipeline (http://ieg4.rccc.ou.edu/mena/) using the recommended Pearson correlation coefficient and a random matrix theory (RMT) modeling for threshold identification [37]. Modules were detected by fast greedy modularity optimization [90]. According to the within-module connectivity (*z*_*i*_) and among-module connectivity (*P*_*i*_), the nodes (ASVs) in a network could be divided into four subcategories, i.e., peripheral nodes, connectors, module hubs and network hubs [37, 91]. Topological properties of 100 random networks with equal numbers of nodes and edges of the real networks were calculated [92]. Network visualization was conducted using the interactive platform Gephi 0.9.2.

## Supplementary information

**Additional file 1: Fig. S1.** Overview map of the River Kaidu catchment showing the sampling sites in upstream tributaries (A01~A08), River Kaidu (B01~B08) and Lake Bosten (C01~C12).

**Additional file 2: Fig. S2.** Rarefaction curves of species richness along the percentage of normalized reads based on the smallest sample (23,965 reads) for samples in the upstream tributaries, the Kaidu River and Lake Bosten. Each vertical bar represents standard error. The curves reach saturation stage with increasing sequencing depth, indicating that the population capture most microbial (bacteria and archaea) members from each sampling type.

**Additional file 3: Fig. S3.** The Spearman correlations between microbial *α*-diversity indices and environmental parameters. Red color means highly positive correlation and blue color means highly negative correlation. The numbers in each plot are the correlation coefficient (ρ) and the significance levels (**P < 0.01; ***P < 0.001). The environmental parameters include concentrations of total suspended solids (TSS), total phosphorus (TP), dissolved oxygen (DO), total dissolved solids (TDS), water temperature (WT), water pH value (pH), dissolved organic carbon (DOC), total nitrogen (TN) and chlorophyll-*a* (Chl-*a*).

**Additional file 4: Table S1.** The amplicon sequence variants (ASVs) table for all sample.

**Additional file 5: Fig. S4.** Taxonomy composition of main microbial communities in each sampling type at **a** phylum-level, **b** class-level and **c** genus-level.

**Additional file 6: Table S2.** The top 10 ASVs in river and lake habitats and their taxonomic classification.

**Additional file 7: Fig. S5.** Mean proportions of predicted functional groups across samples from river habitats (upstream tributaries and mainstream of the Kaidu River, *n* = 15) to lake habitats (Lake Bosten, *n* = 11).

**Additional file 8: Table S3.** Summary of CCA results using forward selection procedure on square root transformed environmental variables.

**Additional file 9: Fig. S6.** Plot showing the distribution of ASVs based on their topological roles. Each symbol represents an ASV from microbial communities in river or lake habitat. The topological role of each ASV was determined according to the scatter plot of within-module connectivity (*zi*) and among-module connectivity (*Pi*). According to the *zi* and *Pi* values, the nodes in a network could be divided into the following four subcategories: (1) peripheral nodes (*zi* < 2.5, *Pi* < 0.62), (2) connectors (*zi* < 2.5, *Pi* ≥ 0.62), (3) module hubs (*zi* ≥ 2.5, *Pi* < 0.62), and (4) network hubs (*zi* ≥ 2.5, *Pi* ≥ 0.62).

**Additional file 10: Table S4.** The top 2 ASVs in the river habitat with their closest 5 relatives in NCBI GenBank database showing the source of them.

**Additional file 11: Fig. S7.** Comparison of principal environmental parameters in the upstream tributaries, the Kaidu River and Lake Bosten. Horizontal bars in the box plots indicate median proportional values. Lower and upper edges of the boxes represent the approximate 1st and 3rd quartiles, respectively. The upper and lower whiskers extend to data no more than 1.5 times the interquartile range from the upper edge and lower edge of the box, respectively. Kruskal-Wallis test was performed to examine differences among the three sampling types with *P*-value presented at the top of each panel. WT, water temperature; EC, electrical conductivity; TDS, total dissolved solids; DO, dissolved oxygen; DOC, dissolved organic carbon; TN, total nitrogen; TP, total phosphorus; Chl-*a*, chlorophyll-*a*; TSS, total suspended solids; BA, bacterial abundance.

## Data Availability

The raw 16S rRNA gene sequences generated in the present study were deposited in the Genome Sequence Archive (GSA) database (http://gsa.big.ac.cn) under accession number CRA001976.
