# Correction to: Contrast diversity patterns and processes of microbial community assembly in a river-lake continuum across a catchment scale in northwestern China

**DOI:** 10.1186/s40793-020-00360-z

**Published:** 2020-07-15

**Authors:** Xiangming Tang, Guijuan Xie, Keqiang Shao, Yang Hu, Jian Cai, Chengrong Bai, Yi Gong, Guang Gao

**Affiliations:** 1 Taihu Laboratory for Lake Ecosystem Research, State Key Laboratory of Lake Science and Environment, Nanjing Institute of Geography and Limnology, Chinese Academy of Sciences, Nanjing, 210008 China; 2 University of Chinese Academy of Sciences, Beijing, 100049 China

**Correction to: Environmental Microbiome (2020) 15:10**

**https://doi.org/10.1186/s40793-020-00356-9**

Following publication of the original article [1], the authors reported an error in Fig. 2; in panel e (‘Fig. 2e’), the observed species abundance distributions and the fitted GS curve have been erroneously omitted.

**Fig. 2:**
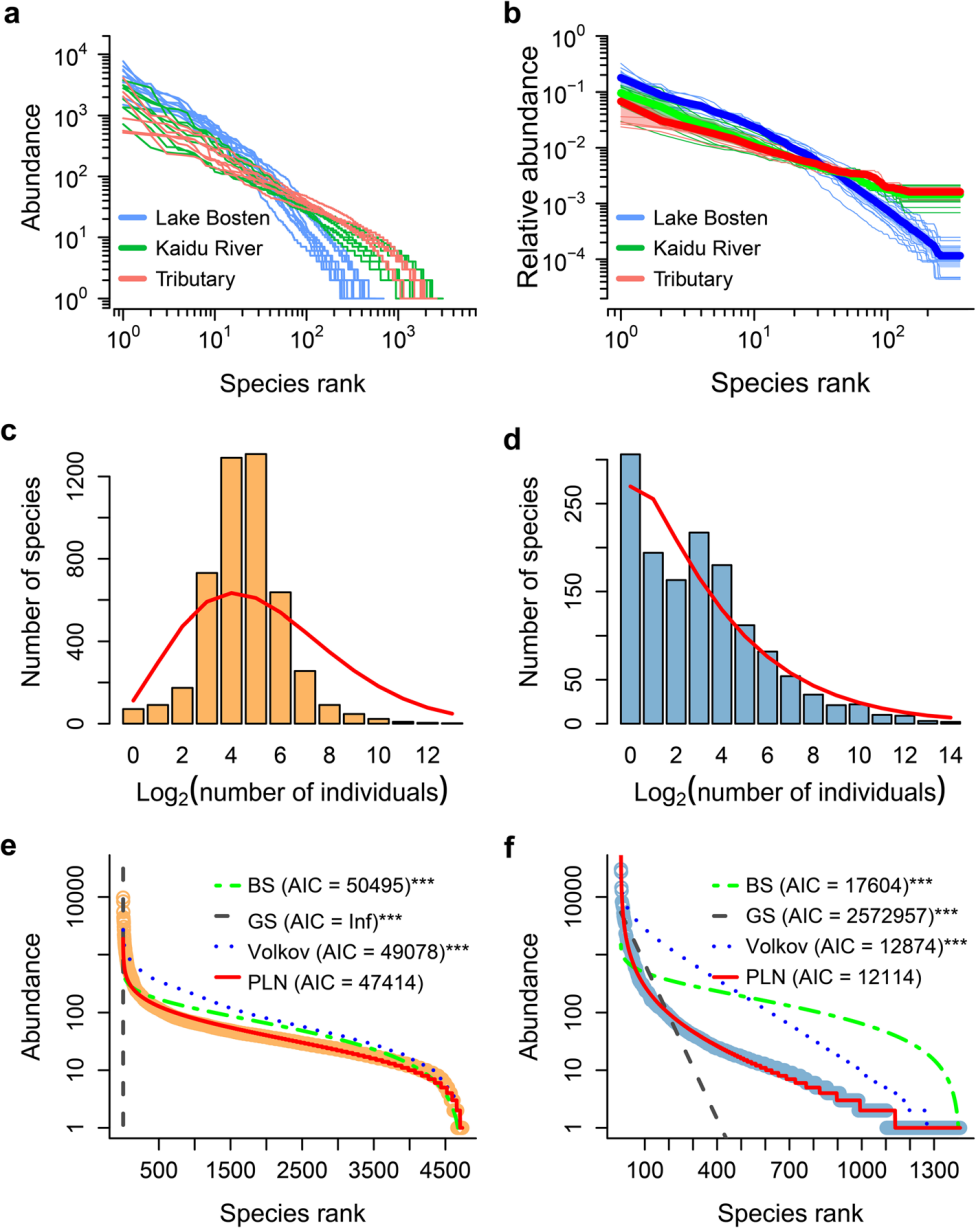
The patterns of species-abundance distributions (SADs) in microbial communities of this study. **a** A rank abundance distribution plot for all samples. **b** Normalized rank abundance distributions (NRADs) with the lowest species (350) sample for samples from upstream tributary (red), River (green) and Lake Bosten (blue). Bold lines and their shaded regions are mean NRADs and the 95% confidence intervals, respectively. SADs of grouped data (binned) from river (**c**, combination of upstream tributary and River Kaidu) and lake (**d**, Lake Bosten) habitats with the predicted values linked as red lines. Observed and fitted SADs for the river **e** and lake **f** microbial communities. Observed values are shown as open circles and fitted models are shown as lines. BS, GS, Volkov and PLN represent broken-stick, geometric-series, Volkov’s neutral community distribution and Poisson log-normal distribution models, respectively. The model was rejected when Kolmogorov-Smirnov (*K-S*) test *P* < 0.05 and the smaller the Akaike’s information criterion (*AIC*) value, the more robust the fit. ^***^K-S test *P* < 0.001. In both habitats, the PLN clearly provide a superior fit

To address this, please find in this correction the corrected figure with the observed species abundance distributions (in orange) and the fitted GS curve (in dark grey) included in ‘e’.
