# Supplementary material for: Contrast diversity patterns and processes of microbial community assembly in a river-lake continuum across a catchment scale in northwestern China
Source: Environ Microbiome. 2020 Apr 25;15:10. doi: 10.1186/s40793-020-00356-9 (PMC8066441; doi:10.1186/s40793-020-00356-9)
Supplement: Supplementary file 1 — Additional file 1: Fig. S1. Overview map of the River Kaidu catchment showing the sampling sites in upstream tributaries (A01~A08), River Kaidu (B01~B08) and Lake Bosten (C01~C12). [file 40793_2020_356_MOESM1_ESM.pdf]

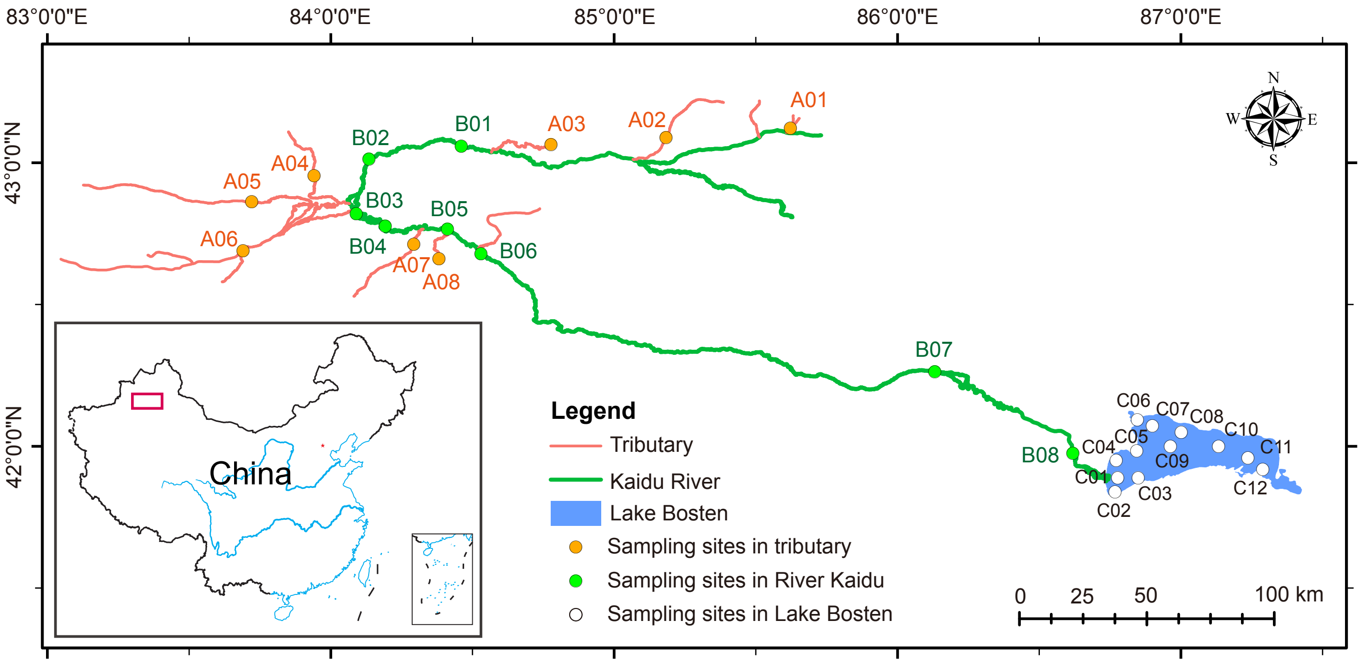

Figure S1. Overview map of the River Kaidu catchment showing the sampling sites in upstream tributaries (A01~A08), River Kaidu (B01~B08) and Lake Bosten (C01~C12).
