# Supplementary material for: Contrast diversity patterns and processes of microbial community assembly in a river-lake continuum across a catchment scale in northwestern China
Source: Environ Microbiome. 2020 Apr 25;15:10. doi: 10.1186/s40793-020-00356-9 (PMC8066441; doi:10.1186/s40793-020-00356-9)
Supplement: Supplementary file 2 — Additional file 2: Fig. S2. Rarefaction curves of species richness along the percentage of normalized reads based on the smallest sample (23,965 reads) for samples in the upstream tributaries, the Kaidu River and Lake Bosten. Each vertical bar represents standard error. The curves reach saturation stage with increasing sequencing depth, indicating that the population capture most microbial (bacteria and archaea) members from each sampling type. [file 40793_2020_356_MOESM2_ESM.pdf]

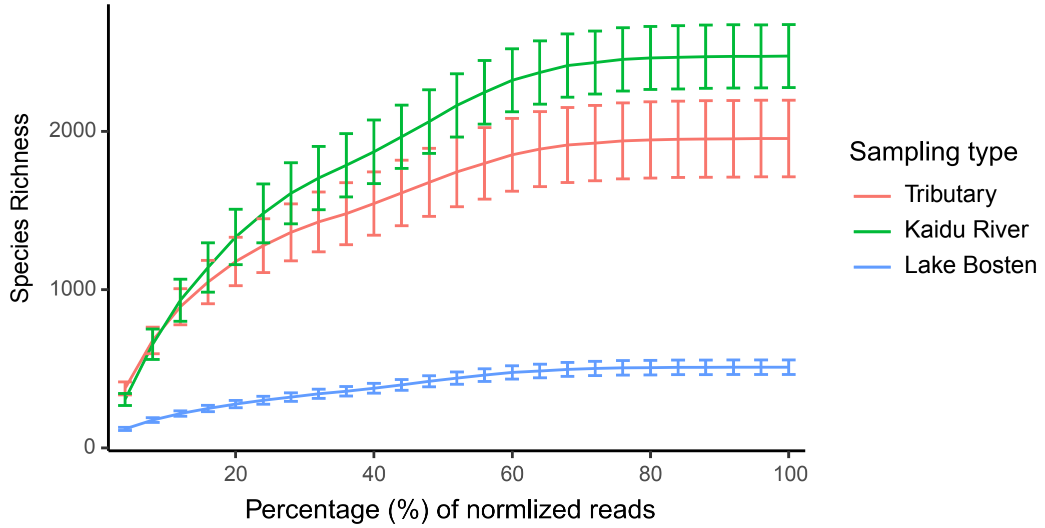

Figure S2. Rarefaction curves of species richness along the percentage of normalized reads based on the smallest sample (23,965 reads) for samples in the upstream tributaries, the Kaidu River and Lake Bosten. Each vertical bar represents standard error. The curves reach saturation stage with increasing sequencing depth, indicating that the population capture most microbial (bacteria and archaea) members from each sampling type.
