# Supplementary material for: Contrast diversity patterns and processes of microbial community assembly in a river-lake continuum across a catchment scale in northwestern China
Source: Environ Microbiome. 2020 Apr 25;15:10. doi: 10.1186/s40793-020-00356-9 (PMC8066441; doi:10.1186/s40793-020-00356-9)
Supplement: Supplementary file 3 — Additional file 3: Fig. S3. The Spearman correlations between microbial α-diversity indices and environmental parameters. Red color means highly positive correlation and blue color means highly negative correlation. The numbers in each plot are the correlation coefficient (ρ) and the significance levels (**P < 0.01; ***P < 0.001). The environmental parameters include concentrations of total suspended solids (TSS), total phosphorus (TP), dissolved oxygen (DO), total dissolved solids (TDS), water temperature (WT), water pH value (pH), dissolved organic carbon (DOC), total nitrogen (TN) and chlorophyll-a (Chl-a). [file 40793_2020_356_MOESM3_ESM.pdf]

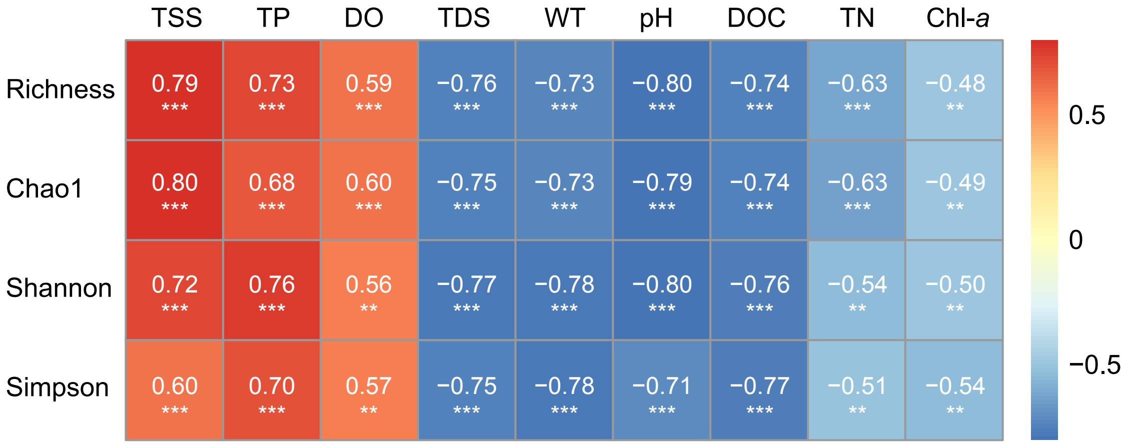

Figure S3. The Spearman correlations between microbial *α*-diversity indices and environmental parameters. Red color means highly positive correlation and blue color means highly negative correlation. The numbers in each plot are the correlation coefficient (ρ) and the significance levels (**$P < 0.01$; ***$P < 0.001$). The environmental parameters include concentrations of total suspended solids (TSS), total phosphorus (TP), dissolved oxygen (DO), total dissolved solids (TDS), water temperature (WT), water pH value (pH), dissolved organic carbon (DOC), total nitrogen (TN) and chlorophyll-*a* (Chl-*a*).
