# Supplementary material for: Contrast diversity patterns and processes of microbial community assembly in a river-lake continuum across a catchment scale in northwestern China
Source: Environ Microbiome. 2020 Apr 25;15:10. doi: 10.1186/s40793-020-00356-9 (PMC8066441; doi:10.1186/s40793-020-00356-9)
Supplement: Supplementary file 5 — Additional file 5: Fig. S4. Taxonomy composition of main microbial communities in each sampling type at a phylum-level, b class-level and c genus-level. [file 40793_2020_356_MOESM5_ESM.pdf]

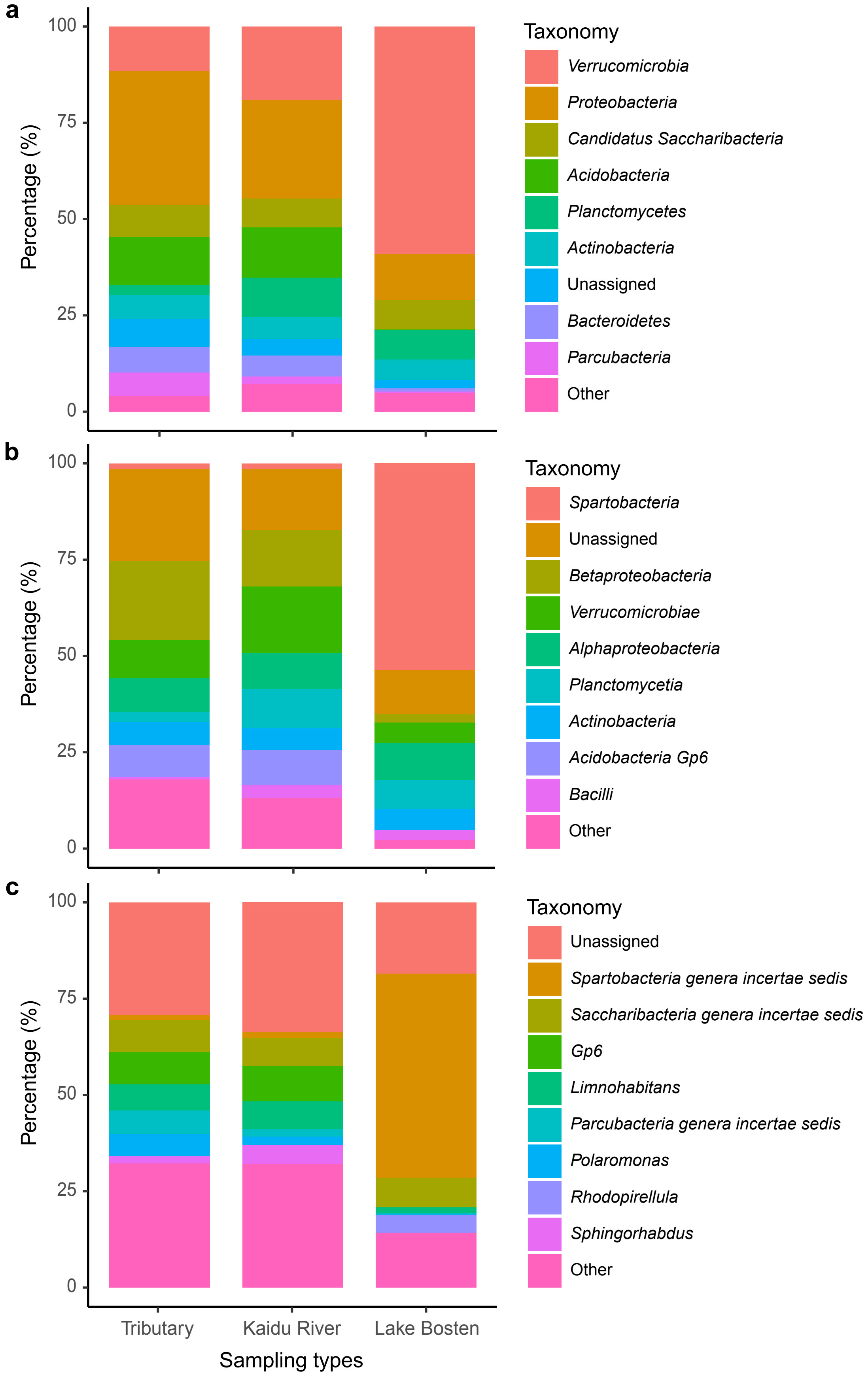

Figure S4. Taxonomy composition of main microbial communities in each sampling type at **a** phylum-level, **b** class-level and **c** genus-level.
