# Supplementary material for: Contrast diversity patterns and processes of microbial community assembly in a river-lake continuum across a catchment scale in northwestern China
Source: Environ Microbiome. 2020 Apr 25;15:10. doi: 10.1186/s40793-020-00356-9 (PMC8066441; doi:10.1186/s40793-020-00356-9)
Supplement: Supplementary file 6 — Additional file 6: Table S2. The top 10 ASVs in river and lake habitats and their taxonomic classification. [file 40793_2020_356_MOESM6_ESM.pdf]

Table S2 The top 10 ASVs in river and lake habitats and their taxonomic classification.

Rank	ASV_ID	Mean abundance (%)		Taxonomy			
		River	Lake	Phylum	Class	Family	Genus
1	ASV_13	2.484	0.001	*Verrucomicrobia*	*Verrucomicrobiae*	*Verrucomicrobiaceae*	Unassigned
2	ASV_5	2.269	0.245	*Verrucomicrobia*	*Verrucomicrobiae*	*Verrucomicrobiaceae*	Unassigned
3	ASV_12	1.650	0.346	*Planctomycetes*	*Planctomycetia*	*Planctomycetaceae*	*Zavarzinella*
4	ASV_18	1.610	0.002	*Proteobacteria*	*Alphaproteobacteria*	*Sphingomonadaceae*	*Sphingorhabdus*
5	ASV_17	1.433	0.100	*Planctomycetes*	*Planctomycetia*	*Planctomycetaceae*	Unassigned
6	ASV_22	1.412	0.111	*Proteobacteria*	*Gammaproteobacteria*	*Pseudomonadaceae*	*Pseudomonas*
7	ASV_7	1.282	0.004	*Proteobacteria*	*Betaproteobacteria*	*Comamonadaceae*	*Limnohabitans*
8	ASV_28	1.151	0.116	*Verrucomicrobia*	*Verrucomicrobiae*	*Verrucomicrobiaceae*	Unassigned
9	ASV_33	1.071	0.008	*Proteobacteria*	*Betaproteobacteria*	*Comamonadaceae*	*Polaromonas*
10	ASV_15	1.010	0.006	*Proteobacteria*	*Betaproteobacteria*	*Comamonadaceae*	*Limnohabitans*
		Lake	River				
1	ASV_1	11.685	0.000	*Verrucomicrobia*	*Spartobacteria*	Unassigned	*Spartobacteria* genera incertae sedis
2	ASV_2	10.780	0.010	*Verrucomicrobia*	*Spartobacteria*	Unassigned	*Spartobacteria* genera incertae sedis
3	ASV_3	5.723	0.004	*Verrucomicrobia*	*Spartobacteria*	Unassigned	*Spartobacteria* genera incertae sedis
4	ASV_4	4.841	0.000	*Proteobacteria*	*Alphaproteobacteria*	SAR11	Candidatus *Fonsibacter*
5	ASV_6	3.232	0.042	*Verrucomicrobia*	*Spartobacteria*	Unassigned	*Spartobacteria* genera incertae sedis
6	ASV_9	3.049	0.002	*Verrucomicrobia*	*Spartobacteria*	Unassigned	*Spartobacteria* genera incertae sedis
7	ASV_8	2.751	0.000	*Verrucomicrobia*	*Spartobacteria*	Unassigned	*Spartobacteria* genera incertae sedis
8	ASV_14	2.685	0.002	*Verrucomicrobia*	*Spartobacteria*	Unassigned	*Spartobacteria* genera incertae sedis
9	ASV_11	2.607	0.000	*Verrucomicrobia*	*Spartobacteria*	Unassigned	*Spartobacteria* genera incertae sedis
10	ASV_10	2.595	0.000	*Verrucomicrobia*	*Spartobacteria*	Unassigned	*Spartobacteria* genera incertae sedis
