# Supplementary material for: Contrast diversity patterns and processes of microbial community assembly in a river-lake continuum across a catchment scale in northwestern China
Source: Environ Microbiome. 2020 Apr 25;15:10. doi: 10.1186/s40793-020-00356-9 (PMC8066441; doi:10.1186/s40793-020-00356-9)
Supplement: Supplementary file 7 — Additional file 7: Fig. S5. Mean proportions of predicted functional groups across samples from river habitats (upstream tributaries and mainstream of the Kaidu River, n = 15) to lake habitats (Lake Bosten, n = 11). [file 40793_2020_356_MOESM7_ESM.pdf]

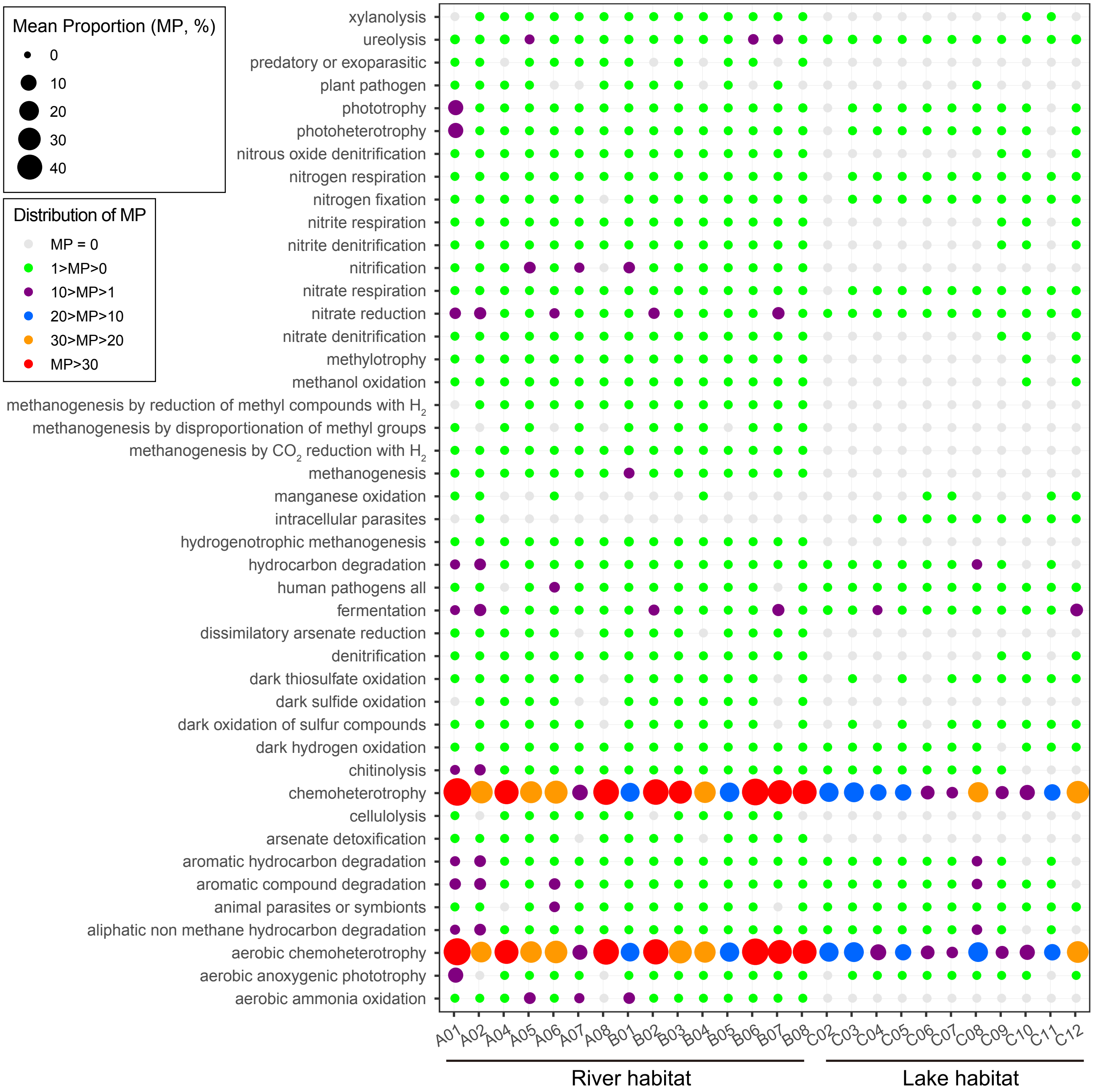

Figure S5. Mean proportions of predicted functional groups across samples from river habitats (upstream tributaries and mainstream of the Kaidu River, *n* = 15) to lake habitats (Lake Bosten, *n* = 11).
