# Supplementary material for: Contrast diversity patterns and processes of microbial community assembly in a river-lake continuum across a catchment scale in northwestern China
Source: Environ Microbiome. 2020 Apr 25;15:10. doi: 10.1186/s40793-020-00356-9 (PMC8066441; doi:10.1186/s40793-020-00356-9)
Supplement: Supplementary file 8 — Additional file 8: Table S3. Summary of CCA results using forward selection procedure on square root transformed environmental variables. [file 40793_2020_356_MOESM8_ESM.pdf]

Table S3 Summary of CCA results using forward selection procedure on square root transformed environmental variables.

Variables	Explains %	Contribution %	pseudo-*F*	*P*	$P_{(adj)}$
TDS	28.4	61.9	9.5	0.001	0.001
TSS	6.2	13.6	2.2	0.001	0.001
WT	5.6	12.2	2.1	0.002	0.003
TN	5.6	12.3	2.2	0.024	0.024

Note: *P* value was generated from permutation test with 999 permutations. $P_{(adj)}$ for multiple testing correction or false discovery rate (FDR).
