# Supplementary material for: Contrast diversity patterns and processes of microbial community assembly in a river-lake continuum across a catchment scale in northwestern China
Source: Environ Microbiome. 2020 Apr 25;15:10. doi: 10.1186/s40793-020-00356-9 (PMC8066441; doi:10.1186/s40793-020-00356-9)
Supplement: Supplementary file 9 — Additional file 9: Fig. S6. Plot showing the distribution of ASVs based on their topological roles. Each symbol represents an ASV from microbial communities in river or lake habitat. The topological role of each ASV was determined according to the scatter plot of within-module connectivity (zi) and among-module connectivity (Pi). According to the zi and Pi values, the nodes in a network could be divided into the following four subcategories: (1) peripheral nodes (zi < 2.5, Pi < 0.62), (2) connectors (zi < 2.5, Pi ≥ 0.62), (3) module hubs (zi ≥ 2.5, Pi < 0.62), and (4) network hubs (zi ≥ 2.5, Pi ≥ 0.62). [file 40793_2020_356_MOESM9_ESM.pdf]

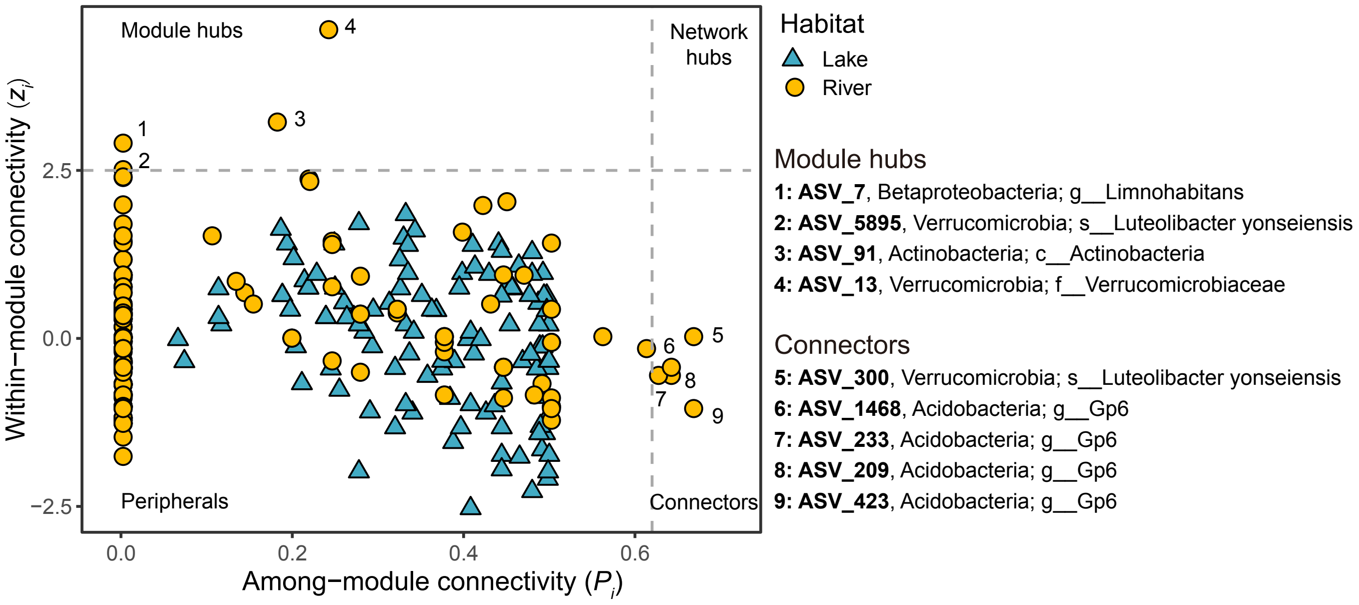

Figure S6. Plot showing the distribution of ASVs based on their topological roles. Each symbol represents an ASV from microbial communities in river or lake habitat. The topological role of each ASV was determined according to the scatter plot of within-module connectivity (z_i) and among-module connectivity (P_i). According to the z_i and P_i values, the nodes in a network could be divided into the following four subcategories: (1) peripheral nodes ($z_i < 2.5$, $P_i < 0.62$), (2) connectors ($z_i < 2.5$, $P_i \geq 0.62$), (3) module hubs ($z_i \geq 2.5$, $P_i < 0.62$), and (4) network hubs ($z_i \geq 2.5$, $P_i \geq 0.62$).
