# Supplementary material for: Contrast diversity patterns and processes of microbial community assembly in a river-lake continuum across a catchment scale in northwestern China
Source: Environ Microbiome. 2020 Apr 25;15:10. doi: 10.1186/s40793-020-00356-9 (PMC8066441; doi:10.1186/s40793-020-00356-9)
Supplement: Supplementary file 10 — Additional file 10: Table S4. The top 2 ASVs in the river habitat with their closest 5 relatives in NCBI GenBank database showing the source of them. [file 40793_2020_356_MOESM10_ESM.pdf]

Table S4 The top 2 ASVs in the river habitat with their closest 5 relatives in NCBI GenBank database showing the source of them.

ASVs	Query Cover	Percentage of Identity	Accession	Source
ASV_13	100%	100%	MG278022.1	Leaf, root, soil
	100%	100%	KF494650.1	Permafrost soil
	100%	100%	JX257578.1	Periphyton
	100%	100%	HF543906.1	Biofilm of stone
	100%	100%	JN232912.1	Reclaimed wastewater
ASV_5	100%	100%	KC683173.1	Periphyton
	100%	100%	KC683164.1	Periphyton
	100%	100%	JN868993.1	Lake Taihu
	100%	100%	FQ660363.1	PAH-contaminated soil
	100%	100%	HQ602857.1	Culture inoculated with sediment
