# Supplementary material for: Contrast diversity patterns and processes of microbial community assembly in a river-lake continuum across a catchment scale in northwestern China
Source: Environ Microbiome. 2020 Apr 25;15:10. doi: 10.1186/s40793-020-00356-9 (PMC8066441; doi:10.1186/s40793-020-00356-9)
Supplement: Supplementary file 11 — Additional file 11: Fig. S7. Comparison of principal environmental parameters in the upstream tributaries, the Kaidu River and Lake Bosten. Horizontal bars in the box plots indicate median proportional values. Lower and upper edges of the boxes represent the approximate 1st and 3rd quartiles, respectively. The upper and lower whiskers extend to data no more than 1.5 times the interquartile range from the upper edge and lower edge of the box, respectively. Kruskal-Wallis test was performed to examine differences among the three sampling types with P-value presented at the top of each panel. WT, water temperature; EC, electrical conductivity; TDS, total dissolved solids; DO, dissolved oxygen; DOC, dissolved organic carbon; TN, total nitrogen; TP, total phosphorus; Chl-a, chlorophyll-a; TSS, total suspended solids; BA, bacterial abundance. [file 40793_2020_356_MOESM11_ESM.pdf]

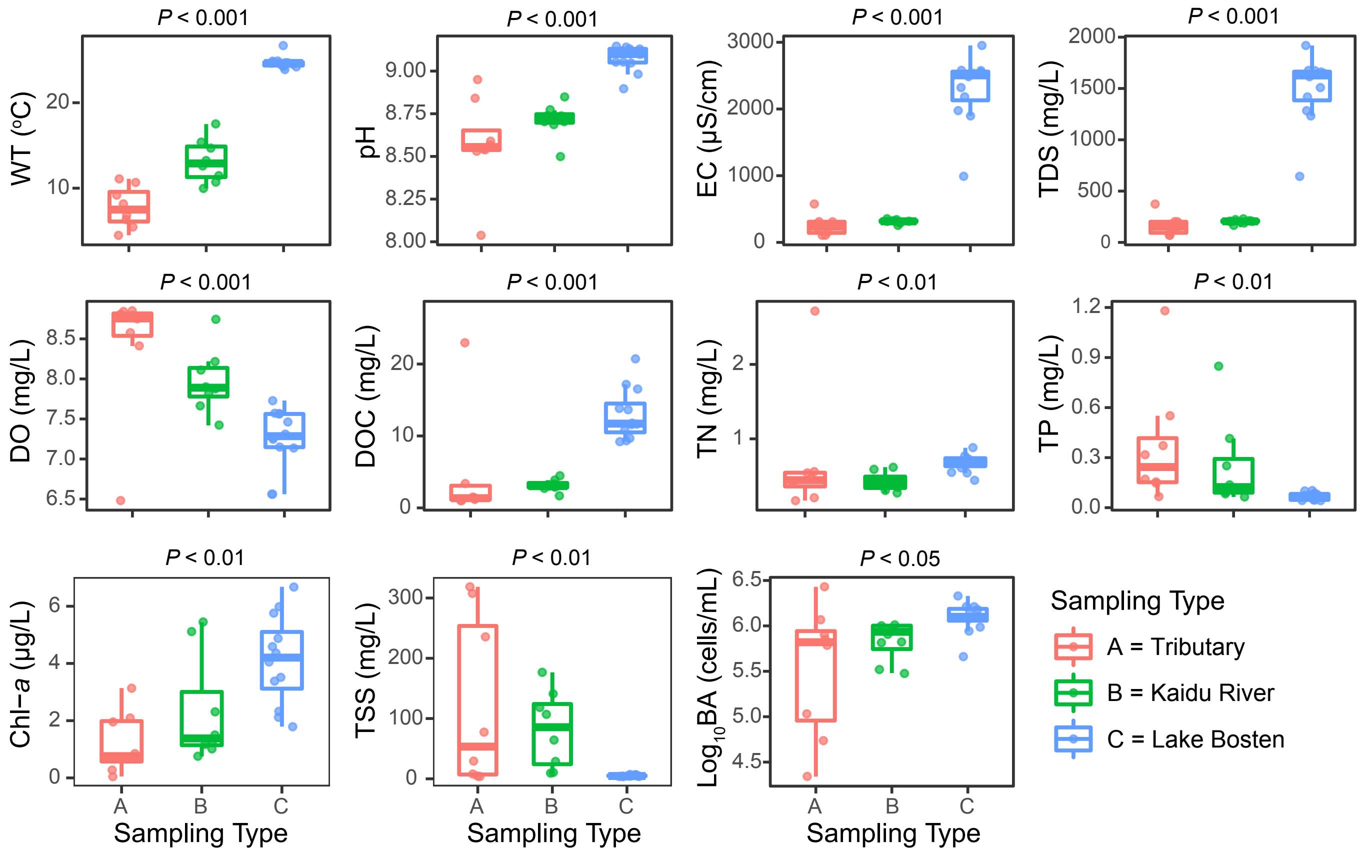

Figure S7. Comparison of principal environmental parameters in the upstream tributaries, the Kaidu River and Lake Bosten. Horizontal bars in the box plots indicate median proportional values. Lower and upper edges of the boxes represent the approximate 1st and 3rd quartiles, respectively. The upper and lower whiskers extend to data no more than 1.5 times the interquartile range from the upper edge and lower edge of the box, respectively. Kruskal-Wallis test was performed to examine differences among the three sampling types with P-value presented at the top of each panel. WT, water temperature; EC, electrical conductivity; TDS, total dissolved solids; DO, dissolved oxygen; DOC, dissolved organic carbon; TN, total nitrogen; TP, total phosphorus; Chl-*a*, chlorophyll-*a*; TSS, total suspended solids; BA, bacterial abundance.
